# Elevated Glutamatergic Compounds in Pregenual Anterior Cingulate in Pediatric Autism Spectrum Disorder Demonstrated by^ 1^H MRS and ^1^H MRSI

**DOI:** 10.1371/journal.pone.0038786

**Published:** 2012-07-27

**Authors:** Anthony Bejjani, Joseph O'Neill, John A. Kim, Andrew J. Frew, Victor W. Yee, Ronald Ly, Christina Kitchen, Noriko Salamon, James T. McCracken, Arthur W. Toga, Jeffry R. Alger, Jennifer G. Levitt

**Affiliations:** 1 Division of Child and Adolescent Psychiatry, Semel Institute for Neurosciences, David Geffen School of Medicine at UCLA, Los Angeles, California, United States of America; 2 Laboratory of Neuroimaging, Department of Neurology, David Geffen School of Medicine at UCLA, Los Angeles, California, United States of America; 3 Ahmanson-Lovelace Brain Mapping Center, Department of Neurology, David Geffen School of Medicine at UCLA, Los Angeles, California, United States of America; 4 Department of Biostatistics, David Geffen School of Medicine at UCLA, Los Angeles, California, United States of America; 5 Department of Radiological Sciences, David Geffen School of Medicine at UCLA, Los Angeles, California, United States of America; 6 Brain Research Institute, David Geffen School of Medicine at UCLA, Los Angeles, California, United States of America; King's College London, United Kingdom

## Abstract

Recent research in autism spectrum disorder (ASD) has aroused interest in anterior cingulate cortex and in the neurometabolite glutamate. We report two studies of pregenual anterior cingulate cortex (pACC) in pediatric ASD. First, we acquired *in vivo* single-voxel proton magnetic resonance spectroscopy (^1^H MRS) in 8 children with ASD and 10 typically developing controls who were well matched for age, but with fewer males and higher IQ. In the ASD group in midline pACC, we found mean 17.7% elevation of glutamate + glutamine (Glx) (*p*<0.05) and 21.2% (*p*<0.001) decrement in creatine + phosphocreatine (Cr). We then performed a larger (26 subjects with ASD, 16 controls) follow-up study in samples now matched for age, gender, and IQ using proton magnetic resonance spectroscopic imaging (^1^H MRSI). Higher spatial resolution enabled bilateral pACC acquisition. Significant effects were restricted to right pACC where Glx (9.5%, *p*<0.05), Cr (6.7%, *p*<0.05), and *N*-acetyl-aspartate + *N*-acetyl-aspartyl-glutamate (10.2%, *p*<0.01) in the ASD sample were *elevated* above control. These two independent studies suggest hyperglutamatergia and other neurometabolic abnormalities in pACC in ASD, with possible right-lateralization. The hyperglutamatergic state may reflect an imbalance of excitation over inhibition in the brain as proposed in recent neurodevelopmental models of ASD.

## Introduction

Proposed abnormal glutamate (Glu) metabolism in autism spectrum disorder (ASD) [1]–[7] is supported by several lines of evidence. Epilepsy is common in ASD [8] and epileptic seizures are propagated by excitatory Glu. Elevated Glu and other excitatory amino acids have been reported in blood serum, plasma, and platelets in ASD [9]–[13]. Post-mortem neuropathology in ASD has found elevated mRNA or protein levels of glutamatergic transporters and neurotransmitter receptors [14]. And finally, ASD has been associated with single-nucleotide polymorphisms (SNPs) in glutamatergic genes, including those coding for transporters [15], metabotropic and ionotropic receptors [3], [16], [17], the enzyme glutamate decarboxylase [18], and the mitochondrial aspartate/glutamate carrier [19]–[26]. The last listed is also supported by neuropathology [27]. These various linkages give ample reason to ask if brain levels of Glu and related metabolites are disturbed in ASD and if such abnormalities have a bearing on clinical presentation. Neuroimaging assays of regional levels of these compounds may help evaluate glutamatergic theories of ASD and inform potential therapies targeting Glu in specific brain structures.

Proton magnetic resonance spectroscopy (^1^H MRS) is a neuroimaging technique that measures in vivo brain Glu safely and non-invasively in children. Thereby, “Glx”, the combined signal for Glu and spectrally overlapping glutamine (Gln), is often more reliably assayed than Glu alone, especially at low field (<3 T). MRS investigations of autistic spectrum disorders have been numerous [28]–[52], but few have reported Glu or Glx [28], [31], [32], [35], [47], [53]. All of these studies detected evidence of glutamatergic abnormalities except Friedman et al.[31], [32], who examined very young (<5 years old) children with ASD. This suggests that further exploration of regional Glu and Glx in the brain in ASD is warranted.

One brain region frequently implicated in ASD, but where glutamatergic metabolites have been little explored [53], is the anterior cingulate cortex. Evidence from several investigative modalities points to involvement of the anterior cingulate in ASD, including neuropathology [54]–[57], structural MRI [58]–[62], fMRI [63]–[67], MRS [33], [42], [46], [53], PET [68], [69], SPECT [70], and EEG evoked potentials [71]. One hypothesis also relates anterior cingulate dysfunction to deficits in joint attention and social orienting in ASD [72]. This plentiful prior work gives reason to search for further abnormalities, perhaps involving the glutamatergic system, in anterior cingulate. Here, we report on two independent studies of glutamatergic neurometabolites in the anterior cingulate cortex in pediatric ASD; the first a pilot study, the second a larger follow-up investigation.

Within the cingulate gyrus, our investigations focused on the pregenual anterior cingulate cortex (pACC) subregion, one of the eight subregions in Vogt's definitive parcellation of the human cingulate cortex [73], [74]. Most of the above-cited neuroimaging studies localized their acquisition or analysis volumes based on the older four-subregion or two-subregion cingulate models. As the eight-subregion model is most consistent with extant neuropathological, neuroimaging, and neurocognitive data, we anticipated that focusing on a subregion within this model would improve odds of detecting Glx effects and would permit more anatomically standardized statement of our results. Moreover, recent neuroimaging investigations, including multimodal MRS-fMRI and combined fMRI and genetic work, of autistic symptoms and autistic traits in healthy subjects have demonstrated focal effects within the pACC, raising the possibilities for finding MRS effects there as well [65], [66], [75], [76].

## Experiment 1

The first experiment was a pilot study targeting possible abnormalities in Glx in ASD in the pACC. The midline (left + right) pACC was sampled using single-voxel MRS.

### Methods

Eight high-functioning subjects with ASD (1 female; mean ± SD age: 11.2±2.6 years, range: 7.8–15.9 years) and 10 typically developing control subjects (5 female; 13.2±2.5 years, 7.4–16.5 years) free of developmental or psychiatric disorder participated (Table 1). Seven subjects with ASD met criteria for autism and one subject met criteria for PDD-NOS according to the Autism Diagnostic Interview Revised (ADI-R) and the Autism Diagnostic Observation Schedule (ADOS) [77]–[78]. These standardized instruments were administered by study personnel trained to reliability by Dr. Catherine Lord's research group. Exclusionary criteria included the presence of major medical or neurologic illness, including epilepsy, and presence of a known genetic syndrome associated with autism such as Fragile X. Control subjects were recruited from local community schools. All subjects were screened for neurological, language, hearing or psychiatric disorders by K-SADS-PL interview with the parent [79]. Exclusion criteria for healthy controls included any lifetime significant medical or Axis I mental disorder. The two subject samples did not differ significantly in age. One subject with ASD was on fluoxetine, two were on methylphenidate, and the remaining subjects were receiving no psychoactive medication at time of scan. No controls were undergoing neuropharmacologic treatment at time of MRS. Mean full-scale IQ (Wechsler Intelligence Scale for Children – WISC) [80] was 90.0±11.5 (74–105) for the sample with ASD and 112.3±18.5 (83–137) for the control sample, a significant difference (p<0.05, independent T-test of rank-transformed data). The study was approved by the UCLA Human Subjects Review Board and we obtained written, informed consent from all subjects or from the subjects' parents or guardians.

**Table 1:** Experiment 1: Subject group characteristics.

group	gender	age, years	full-scale IQ	medication
***ASD***	7 male, 1 female	11.2±2.6 (7.8–15.9)	**90.0±11.5** * (74–105)	1 fluoxetine, ***2*** methylphenidate
***Typically developing***	5 male, 5 female	13.2±2.5 (7.4–16.5)	112.3±18.5 (83–137)	None

* *p*<0.05 *vs.* controls; values for age and IQ are group mean ± standard deviation (range).

**Figure 1:**
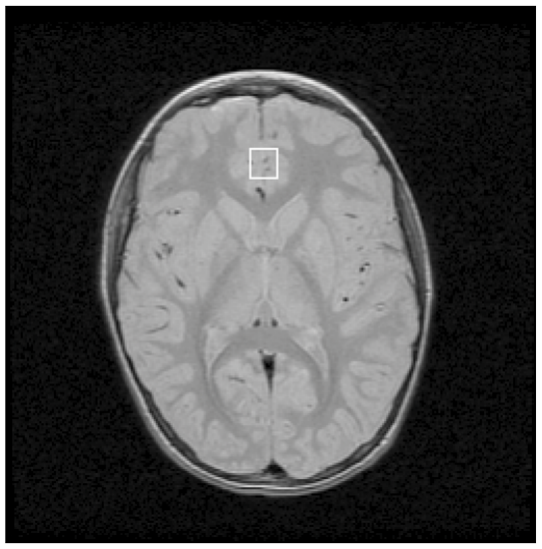
Axial-oblique spin-echo MRI of the brain at level of the basal ganglia showing position of midline pregenual anterior cingulate (**pACC**) **proton magnetic resonance spectroscopy voxel** (***white box***)**. Experiment** **1.**

MRI and single-voxel ^1^H MRS were acquired contemporaneously at 1.5 T on a GE Signa 5× system with a quadrature head coil. After sagittal scout, a whole-brain axial fast spin-echo MRI yielding proton density-weighted images and a coronal T1-weighted MRI (brain stem and forward) were acquired. These two MRI volumes were used to position single-voxel water-suppressed point-resolved spectroscopy (PRESS) ^1^H MRS (GE PROBE, repetition time [TR]  = 1500 ms, echo-time [TE]  = 25 ms, number of excitations  = 256) in midline pACC (Fig. 1) in order to assess potential group differences in local metabolite levels. Voxel position and volume (2.4–3.6 cc, 12–15 mm on a side) were varied to maximize gray-matter content. This procedure was applied identically for subjects with ASD and controls by operators blinded to subject diagnosis. The pACC was identified on MRI as the cingulate cortex directly rostral to the genu of the corpus callosum where the posterior wall of the MRS voxel was placed. Each scan was examined post-acquisition. When quality was low, the scan was repeated. Total session time was 1–1.5 hours. One subject with ASD was sedated with intravenous propofol during acquisition. Results for this subject did not differ markedly from those of the other subjects with ASD. In a separate MR session, a sagittal whole-brain volumetric acquisition was performed using a spoiled gradient recalled echo (SPGR) sequence (TR  = 24 ms, TE  = 9 ms, number of excitations  = 2, in-plane resolution  = 0.94×0.94 mm^2^, partition thickness  = 1.2 mm), yielding T1-weighted images used for MRI tissue-segmentation.

Operators were blinded to subject identity during MRS and MRI post-processing. MRS PROBE data were processed automatically with LCModel [81]. LCModel yielded absolute levels in Institutional Units (IU) scaled to water and accounting for variable voxel volume for five major metabolite peaks. The first peak represented the sum of the (very closely overlapping) signals for N-acetyl-aspartate (NAA) and N-acetyl-aspartyl-glutamate (NAAG); the sum is referred to as “total NAA”, abbreviated “tNAA”. The second peak, “Glx”, was for the sum of glutamate and glutamine. The third was for the sum of creatine and phosphocreatine, together abbreviated “Cr”. The fourth was for the sum of the choline-containing compounds phosphocholine, glycerophosphocholine, choline proper, and acetylcholine, together abbreviated “Cho”. The final peak was for the sugar myo-inositol (“mI”). Numerous weaker resonances, in particular lipids and macromolecules, were included in the fit. Analyses were restricted to spectra with linewidth (full-width at half-maximum) ≤0.10 ppm and with signal-to-noise ratio ≥5 and to metabolite peaks that passed the widely applied standard LCModel automated quality control criterion SD ≤20%, supplemented by operator inspection. SPGR-derived T1-weighted MRI volumes were tissue-segmented into gray matter, white matter, and CSF whole-brain component volumes [82] then co-registered into the axial plane of the fast spin-echo MRI, which was already in register with the MRS voxels. A home-written software program in the Interactive Data Language (IDL; Research Systems Inc., Boulder, CO) then extracted volume% gray matter, white matter, and CSF in each MRS voxel. LCModel absolute metabolite levels were then corrected for volume% CSF. Metabolite levels were not corrected for T1- or T2-relaxation.

Given the small number of subjects, data were rank-transformed to provide for non-parametric statistical analyses. MRS voxel tissue composition (volume% gray matter, white matter, CSF) was compared between groups using independent T-test of rank-transformed data. CSF-corrected levels of each metabolite were compared between groups using analysis of covariance (ANCOVA) of rank-transformed data with subject gender as covariate. At sites where tissue composition differed significantly between groups, the relevant volume% variable(s) were added as covariates to ANCOVA. Criterion for statistical significance was p<0.05, Bonferroni-corrected for multiple comparisons with numbered a priori hypotheses. Effects were hypothesized on metabolites in the following order: 1. Glx, 2. tNAA, 3. Cr, 4. Cho, 5. mI.

### Results and Discussion

MRS voxel volume% ranged 74.1–93.9% for gray matter, 3.0–20.4% for white matter, and 0.1–13.7% for CSF (Table 2). Volume% white matter in pACC was significantly lower in subjects with ASD (6.4±2.2%) than in controls (10.8±4.2%; p<0.05); otherwise tissue composition did not differ significantly between groups.

**Table 2:** Experiment 1: Voxel tissue content and proton magnetic resonance spectroscopy (^1^H MRS) metabolite levels in midline pregenual anterior cingulate cortex (pACC) voxel.

	*ASD*		Healthy Control	
	mean ± SD	range	mean ± SD	range
**Vol% GM**	88.9±4.1	83.3–93.9	83.9±5.6	74.1–89.6
**Vol% WM**	**6.4±2.2** *	3.0–10.2	10.8±4.3	4.4–20.4
**Vol% CSF**	4.7±3.1	1.8–10.7	5.1±4.4	0.1–13.7
**tNAA**	7.4±1.6	4.6–9.7	7.5±1.1	6.4–9.5
**Glx**	**17.0±1.4** *	14.7–19.2	14.5±3.0	9.4–18.8
**Cr**	**4.4±0.8** *	2.8–5.4	5.6±0.7	4.7–6.7
**Cho**	1.1±0.2	0.8–1.4	1.2±0.3	0.9–1.9
**mI**	3.7±0.4	3.3–4.5	4.4±0.9	3.0–5.7

* *p*<0.05 *vs.* controls controls analysis-of-covariance covarying gender and vol% WM on rank-transformed (non-parametric) data, Bonferroni-corrected for multiple comparisons with *a priori* hypotheses.

SD  =  standard deviation, GM  =  gray matter, WM  =  white matter, CSF  =  cerebrospinal fluid.

tNAA  =  *N*-acetyl-aspartate + *N*-acetyl-aspartyl-glutamate, Glx  =  glutamate + glutamine, Cr  =  creatine + phosphocreatine, Cho  =  choline compounds, mI  =  *myo*-inositol.

**Figure 2:**
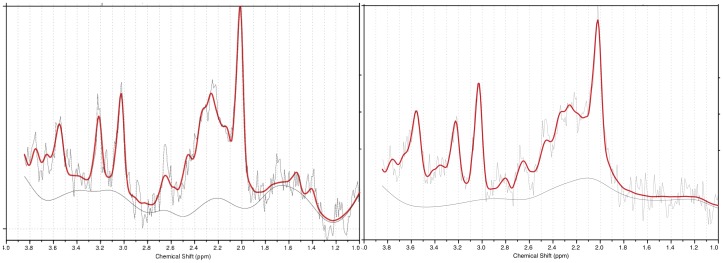
Raw (*light gray*), fit (*heavy solid red*), and baseline (*solid gray*) LCModel (Provencher 2001) output spectra for proton magnetic resonance spectroscopy voxels placed in midline pregenual anterior cingulate (pACC) in study subject with *ASD* (*left*) and control subject (*right*). Note larger Glx peak (2.0–2.5 ppm) in subject with *ASD*. Seven of 8 subjects with ***ASD*** exhibited Glx levels above the control mean (Fig. 3). Experiment 1.

**Figure 3:**
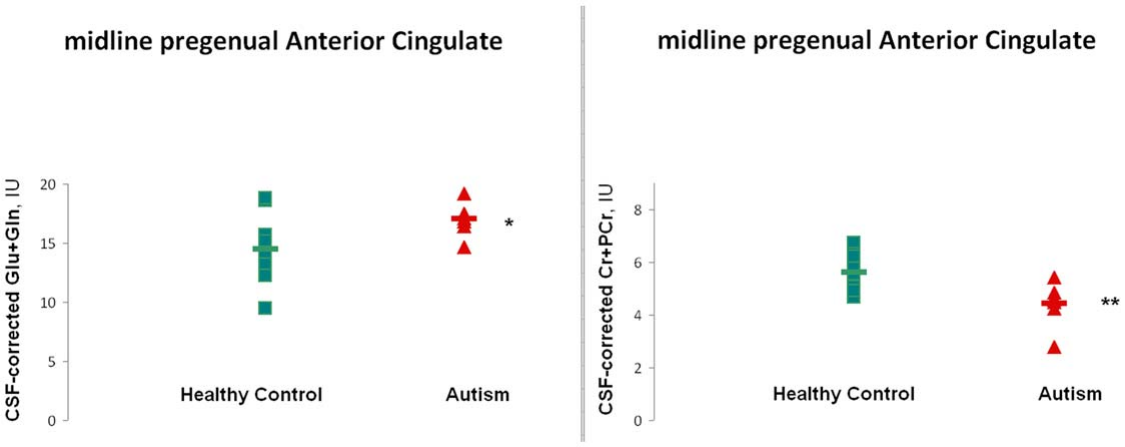
LCModel-derived absolute metabolite levels in Institutional Units (IU) corrected for voxel CSF-content for subjects with *ASD* (*red triangles*) and healthy controls (*green squares*). *Horizontal bars* denote group means. Values are shown for glutamate + glutamine (“Glx”; *left*) and creatine + phosphocreatine (*right*) in pACC. Note elevated Glx and deptressed Cr + PCr in subjects with ***ASD***. *p<.05, **p<.01 (independent T-test following ANCOVA of rank-transformed, therefore non-parametric data.) Experiment 1.

Fig. 2 shows sample LCModel-fitted ^1^H MR spectra for a subject from the sample with ASD and a control subject. To the naked eye the Glx peak is larger in the subject with ASD than in the control. High Glx levels were also seen in most of the other subjects with ASD (Fig. 3). Table 2 lists group-mean CSF-corrected LCModel-derived metabolite values in pACC. For subjects with ASD vs. typically-developing controls, ANCOVA covarying for gender and volume% white matter found significantly higher Glx (17.7%; p<0.05) and significantly lower Cr (21.2%; p<0.05; Fig. 3). No significant effects were found for any other metabolite. Glx was above the control mean for 7 of 8 subjects with ASD. Upon removing subjects on medication, the single-voxel MRS Glx elevation in pACC was still significant (23.2%, p<0.05).

This pilot single-voxel MRS investigation of glutamatergic metabolites in pACC yielded two findings: 1) Glx was consistently higher in subjects with ASD than in typically-developing control subjects and 2) Cr was lower in subjects with ASD than in controls. These findings suggest that abnormal physiology of the anterior cingulate in ASD may include disturbances in Glu or Cr neurometabolism and signal the pACC as one locus of abnormality within the cingulate gyrus.

The first finding was elevated Glx ( =  Glu + Gln) in the pACC. This supports the notion of glutamatergic disturbances in ASD [1]–[7] and favors proposals of hyper- as opposed to hypoglutamatergia, at least in the pACC. Glu is normally the larger component of the Glx signal and is present in tissue in both neurotransmitter and metabolic pools, which cannot be distinguished by in vivo ^1^H MRS [83]. Mechanisms that might increase the combined Glu + Gln  =  Glx concentration include greater pre-synaptic vesicular release of Glu and co-localized NAAG [84], faster breakdown of NAAG into NAA and Glu [85], and net production rather than consumption of Glu by the Krebs Cycle in neurons and astrocytes [86], slower conversion of Gln to GABA [87], and net production rather than consumption of Gln by the Krebs Cycle. Moreover, Gln is exported from the astrocyte by the SNAT3 [88] neutral amino acid membrane transporter, crosses extracellular fluid, and is taken into the neuron by the SNAT1 transporter [89]. Slowing of this Gln transport resulting in longer residence times in the Glu-Gln Cycle [86], [90], [91] would also lead to higher effective Glx levels. Consequences of chronic excess Glx could include abnormal development, ongoing excitotoxic cell damage (particularly in the case of Glu in the neurotransmitter pool), and inefficient utilization of cell energy.

The second finding was diminished Cr in the pACC. Creatine and phosphocreatine are principal reactants in the cellular buffer for maintenance and rapid mobilization of ATP energy [92]–[94]. This Cr deficit, along with the above Glx excess and previously demonstrated ^18^FDG-PET abnormalities in ASD [95], may reflect locally aberrant cell-energy metabolism.

Results will be compared, in conjunction with those of Experiment 2, to previous MRS investigations in a combined discussion below.

This pilot investigation had a small number of subjects. Subjects with ASD were compared to a control group that was not well matched for gender or IQ. Three subjects with ASD were receiving regular psychotropic medication and one underwent sedation during the MRS scan; no controls underwent such pharmacologic treatments. Left and right pACC were sampled together in a single MRS voxel; that voxel contained, on average more white matter in subjects with ASD than in controls (although vol% white matter was under 10% in most subjects and was covaried for statistically). Despite these limitations, Experiment 1 gives a strong indication (7 of 8 subjects) of above-normal glutamatergic compounds in the pACC in ASD. Experiment 2 attempted to address some of the above limitations.

## Experiment 2

The second experiment was a larger study that again targeted Glx in ASD in the pACC. This time, the higher spatial resolution of the proton magnetic resonance spectroscopic imaging (^1^H MRSI) technique allowed us to examine left and right pACC separately, and therefore permitted us to investigate any lateralized effects on glutamatergic metabolites in this region. MRSI furthermore enabled us to investigate the possibility that results of Experiment 1 were influenced by white-matter intrusion into the MRS voxel by additionally obtaining metabolite levels in the prefrontal white-matter regions laterally adjacent to left and right pACC. Subjects with ASD were now compared to typically developing control subjects who were matched for age, gender, and IQ. Based on the results of Experiment 1, we hypothesized elevated Glx and diminished Cr in ASD in pACC, without any preference for the left or right hemisphere.

### Methods

Twenty-six high-functioning subjects with ASD (7 female; mean ± SD age: 10.2±3.3 years, range: 6.1–17.5 years) and 16 typically developing control subjects (5 female; 11.8±3.0 years, 7.3–16.6 years) participated (Table 3). No subject in Experiment 2 was also a subject in Experiment 1. As in Experiment 1, exclusionary criteria included the presence of major medical or neurologic illness, including epilepsy, and presence of a known genetic syndrome associated with autism such as Fragile X. Subjects with ASD were recruited from the Autism Genetic Research Exchange (AGRE) and were included only if they had met criteria for ASD based upon the Autism Diagnostic Interview-Revised (ADI-R) [77] interview and Autism Diagnostic Observation Schedule administered by AGRE personnel. All subjects met criteria for autism according to the ADI-R and seventeen met criteria for autism and eight for ASD according to the ADOS. Five subjects with ASD were being treated with stimulants (2 methylphenidate, 2 dextroamphetamine); 1 was being treated with a selective norepinephrine reuptake inhibitor (atomoxetine); 1 was being treated with an anti-convulsant (levetiracetam); 2 were being treated with anti-depressants (1 fluoxetine, 1 fluvoxamine); and 1 was being treated with an anti-psychotic (risperidone). However, no controls were undergoing neuropharmacologic treatment at time of MRS. Control subjects were recruited from local community schools. Exclusion criteria for healthy controls included any lifetime significant medical or Axis I mental disorder based upon the KSADS or the Diagnostic Interview Schedule for Children (C-DISC-4, Shaffer et al., 2000) interview with the parent [96], with the following exceptions. As 4 of the ASD subjects were being treated with stimulant medications, we included one control subject meeting criteria for inattentive attention-deficit disorder (9 inattentive symptoms on the DISC-IV) and four subjects with 1–2 inattentive symptoms and 2–3 hyperactive symptoms as assessed by the DISC-IV in the control group. The samples did not differ significantly in age. Mean full-scale IQ, assessed using either the Weschler Intelligence Scale for Children (Weschler 1991) or the Weschler Adult Intelligence Scale or (WASI) (Wechsler Abbreviated Scale of Intelligence – WASI) [97], was 106.6±13.5 (76–142) for the sample with ASD and 101.2±13.6 (65–118) for the control sample. This was not a significant difference. One control subject had full-scale IQ 65 and therefore met a formal criterion for mental retardation. This was an otherwise normally functioning subject who did not qualify for any other psychiatric or developmental disorder. Exclusion of this subject did not change the study results. All other participants in both groups had full-scale IQ >75. The study was approved by the UCLA Human Subjects Review Board and we obtained written, informed consent from all subjects or from the subjects' parents or guardians.

**Table 3:** Experiment 2: Subject group characteristics.

group	gender	age, years	full-scale IQ	medication
***ASD***	19 male, 7 female	10.2±3.3 (6.1–17.5)	106.6±13.5 (76–142)	5 stimulants, 1 anti-convulsant, 2 anti-depressants, 1 anti-psychotic
***Typically developing***	11 male, 5 female	11.8±3.0 (7.3–16.6)	101.2±13.6 (65–118)	none

values for age and IQ are group mean ± standard deviation (range).

**Figure 4:**
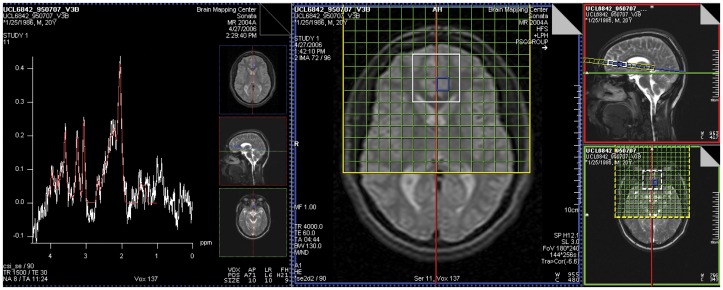
(*Right three panels*) MRI sections showing positioning of axial-oblique (parallel to genu-splenium line in sagittal plane) proton magnetic resonance spectroscopic imaging (^1^H MRSI, TR/TE  = 1500/30 ms) slab (*green grid*). The PRESS acquisition volume (*white box*) sampled bilateral pregenual anterior cingulate cortex (pACC, posterior mesial voxels), mesial superior frontal cortex (anterior mesial voxels) and laterally adjacent white matter (lateral voxels). (*Left panel*) sample spectrum from left pACC voxel selected in blue. The *white trace* depicts the raw data, the *red trace* is a summary fit provided by the Siemens Sonata software. This is superseded by a definitive fit provided by LCModel. An appreciable Glx peak is evident. Experiment 2.

MRI and multi-voxel ^1^H MRSI were acquired contemporaneously at 1.5 T on a Siemens Sonata system with quadrature head coil. No subjects were sedated at time of scan. After initial localizer, an axial-oblique whole-brain double turbo spin-echo (DTSE) structural MRI was acquired oriented parallel to the genu-splenium line as seen in the sagittal plane. This was followed by a sagittal T1-weighted high-resolution whole-brain SPGR structural MRI volume (TR/TE  = 25/11 ms, NEX  = 1, partition thickness  = 1.2 mm contiguous, in-plane resolution  = 1×1 mm^2^). Water-suppressed ^1^H MRSI (PRESS, TR/TE  = 1500/30 ms, NEX  = 8, slab thickness  = 9 mm, in-plane resolution  = 11×11 mm^2^; Fig. 4) was acquired in the plane of the DTSE. The “PRESS box” – acquisition volume from which usable spectra could be obtained – measured 4×4 voxels in cross-section in every subject. The nominal voxel size was 9 mm ×11 mm ×11 mm ≈ 1.1 cc. Identical voxel positioning procedures were employed for subjects with ASD and controls by operators blinded to subject diagnosis. The pACC PRESS box straddled the longitudinal midline and was positioned with its posterior end at the rostrum of the corpus callosum, set just far back enough to prevent the anterior end of the box from contacting extracranial tissue. The slab was also centered dorsoventrally about the callosal rostrum. The lateral-mesial (left-right) position of the grid was moved to minimize differences in voxel composition. In particular, where subject anatomy permitted, the gridlines were positioned such that voxels lay entirely in left or entirely in right pACC and also such that gray-matter content of voxels generally was maximized. Thus, posterior voxels of this box sampled pACC and anterior voxels sampled mesial superior frontal cortex. Lateral voxels sampled prefrontal white matter. This PRESS scan was immediately followed by non-water-suppressed PRESS MRSI (NEX  = 1, otherwise identical parameters). Finally, the whole-brain SPGR was repeated.

Operators were blinded to subject identity during MRI and MRSI post-processing. Offline, for each subject, the two whole-brain SPGR volumes were coregistered to each other and averaged together to enhance signal-to-noise ratio. The averaged SPGR was then manually edited to remove non-brain tissue. Automated programs were used to correct for magnetic field inhomogeneities [98], realign the images into a standard orientation using rigid-body transformations [99], and segment the volume into whole-brain masks representing gray matter, white matter, and CSF using a partial-volume correction method [100]. The masks were reverse-transformed to the subject's native space and binarized.

Siemens MRSI data were processed automatically with LCModel [81]. LCModel yielded absolute levels in Institutional Units (IU) scaled to water for the five major metabolite peaks. Numerous weaker resonances, in particular lipids and macromolecules, were included in the fit.

The UCLA MRSI Voxel Picker (MVP) version 1.0 software suite [101], [102] was used for MRI/MRSI co-processing. The averaged SPGR volume and its gray-matter, white-matter, and CSF component masks were imported into MVP together with the MRSI raw data file and LCModel output. MVP reconstructed the subject's SPGR and displayed it in a guided user interface (GUI) in register with the corresponding MRSI PRESS volume in its plane-of-acquisition. MVP similarly reconstructed the whole-brain gray-matter, white-matter, and CSF binary masks; computed the volume% gray matter, white matter, and CSF in each MRSI voxel; and corrected the LCModel-derived levels of each metabolite for voxel CSF content. Metabolite levels were not corrected for T1- or T2-relaxation. Quality control of MRSI spectra was also implemented automatically by MVP, supplemented by operator inspection. Only spectra that showed a linewidth ≤0.1 ppm and a signal-to-noise ratio ≥3 were used for subsequent analysis. Furthermore, within each voxel, MVP retained only those metabolite values that LCModel considered reliable (SD ≤20%). Voxels were selected by the operator on the MVP GUI in left and right hemispheres for pACC and for adjacent left and right prefrontal white matter. Within each structure, MVP averaged together the metabolite levels and tissue composition for all voxels that contained ≥50 vol% gray matter (≥70 vol% white matter for the white-matter regions), which satisfied the foregoing quality-control criteria.

Data were rank-transformed for non-parametric statistical analyses. MRSI voxel tissue composition (volume% gray matter, white matter, CSF) was compared between groups using independent T-test. To account for multiple comparisons, omnibus testing of CSF-corrected metabolite levels was performed prior to between-group regional comparisons. In particular, for each metabolite a repeated-measures ANCOVA (R-ANCOVA) was performed with Hemisphere (two levels: left, right) as within-subjects factor and Group (two levels: autism, control) as between-subjects factor. Tissue composition was included as covariate in the event of significant differences between groups. For metabolites for which R-ANCOVA yielded a significant main effect or interaction involving Group, post-hoc ANCOVA was performed comparing the between-group metabolite levels in each individual region, with volume% tissue composition included as covariate as appropriate.

As a further test of the possible influence of white-matter intrusion on pACC results, metabolite levels were compared between groups in adjacent left and right prefrontal white matter using independent T-test, to see if the same metabolite effects were present as in the cortex. Criterion for statistical significance for all tests was p<0.05.

### Results and Discussion

MRSI voxel tissue compositions are listed in Table 4. In both left (p<0.0005) and right (p<0.01) pACC volume% gray matter was significantly higher in subjects with ASD than in controls. Contrary to expectation, mean voxel volume% gray matter was 10–20% lower and mean voxel volume% white matter was 5–10% higher than the corresponding values in Experiment 1. The Experiment 1 strategem of varying MRS voxel dimensions was apparently more effective than using smaller MRSI voxels of a fixed size in maximizing gray-matter content. Based on these findings, volume% gray and volume% white matter were included as covariates in between-group comparisons of metabolite levels.

**Table 4:** Experiment 2: Voxel tissue content and proton magnetic resonance spectroscopic imaging (^1^H MRSI) metabolite levels in bilateral pregenual anterior cingulate cortex (pACC) voxel.

	ASD		Healthy Control	
	mean ± SD	range	mean ± SD	range
	*Left pregenual Anterior Cingulate Cortex*			
**vol% GM**	77.7±3.8***	69.5–90.2	67.6±9.5	51.9–85.5
**vol% WM**	13.2±5.4	5.1–23.4	17.2±8.7	8.7–41.9
**vol% CSF**	9.1±5.6	1.6–19.1	8.6±6.7	0.6–21.2
**tNAA**	7.7±1.0	5.6–9.2	7.0±1.0	4.6–8.3
**Glx**	13.6±1.8	10.0–17.6	13.4±1.5	11.5–17.3
**Cr**	5.3±0.9	3.6–6.9	5.0±0.7	3.9–6.2
**Cho**	1.5±0.3	1.1–2.1	1.4±0.2	1.0–1.8
**mI**	4.2±0.7	2.8–5.5	3.9±0.8	2.4–5.2
	*Right pregenual Anterior Cingulate Cortex*			
**vol% GM**	75.9±3.5**	69.4–82.8	70.2±8.7	58.0–90.3
**vol% WM**	14.8±4.4	6.9–24.6	18.7±12.0	2.8–37.7
**vol% CSF**	8.9±3.8	2.8–16.9	10.4±7.1	0.7–22.1
**tNAA**	7.6±1.0**	5.1–10.0	6.9±1.4	5.4–9.2
**Glx**	**14.5±2.2** *	9.2–19.1	13.2±2.6	9.5–17.5
**Cr**	**5.4±0.9** *	3.9–7.3	5.0±1.0	3.9–7.1
**Cho**	1.5±0.3	1.0–2.3	1.4±0.3	1.1–1.9
**mI**	4.1±0.7	2.7–5.5	3.9±1.1	2.9–6.6

* *p*<0.05 *vs.* controls ANCOVA following omnibus R-ANCOVA both covarying vol% GM and WM on rank-transformed (non-parametric) data. Abbreviations as in Table 2.

**Figure 5:**
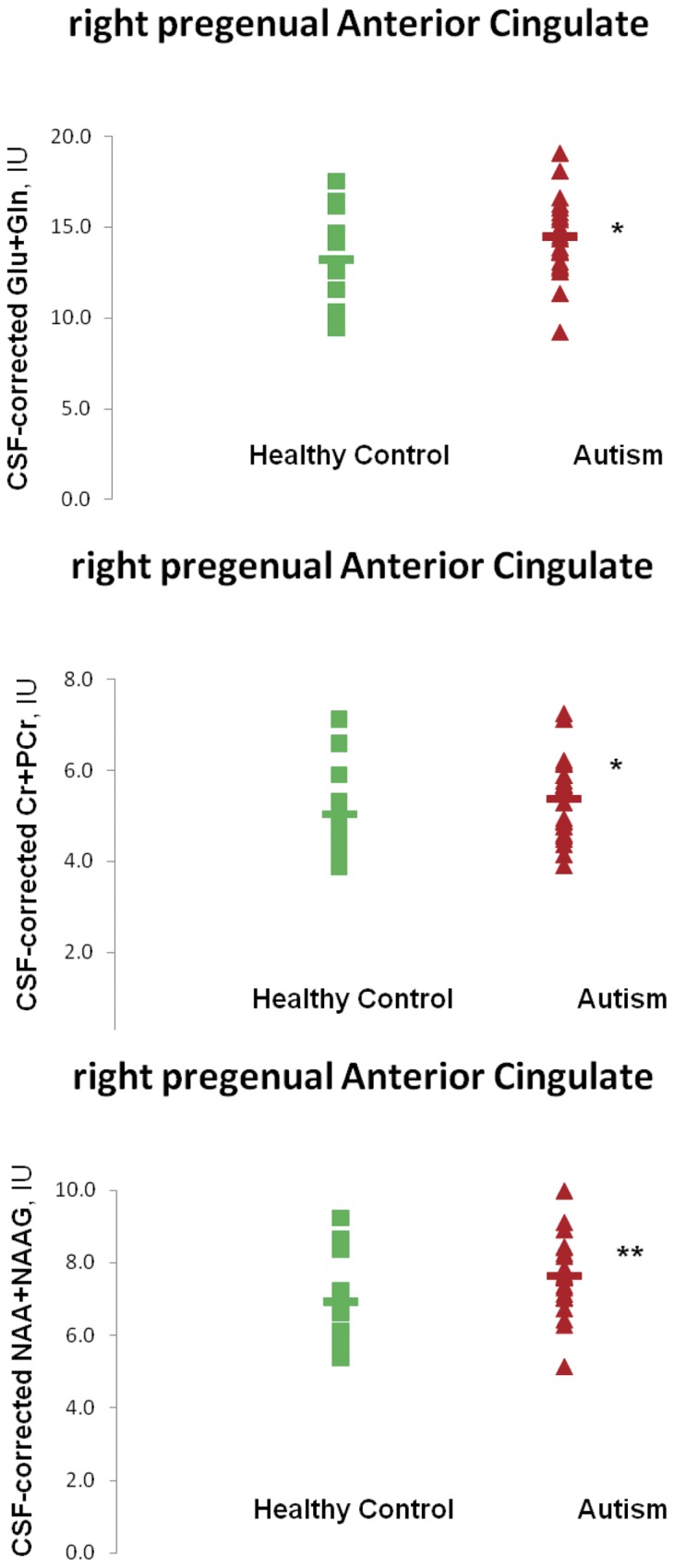
LCModel-derived absolute metabolite levels in Institutional Units (IU) corrected for voxel CSF-content for subjects with *ASD* (*red triangles*) and healthy controls (*green squares*) in right pACC. *Horizontal bars* denote group means. Values are shown for glutamate + glutamine (“Glx”; *top*), creatine + phosphocreatine (*middle*), and *N*-acetyl-aspartate + *N*-acetyl-aspartyl-glutamate. Note elevations of all three metabolites in subjects with ***ASD***. *p<0.05, **p<0.01 (ANCOVA following R-ANCOVA of rank-transformed, therefore non-parametric data.) Experiment 2.

Fig. 4 shows a sample MRSI spectrum from left pACC. An appreciable Glx peak is visible next to the tNAA peak at 2.01 ppm. Table 4 lists group-mean CSF-corrected LCModel-derived metabolite values in left and right pACC. Omnibus R-ANCOVA for pACC covarying volume% left and right pACC gray matter and white matter indicated significant main effects of Group for tNAA (F(1,26) = 5.4, p<0.05) and Cr (F(1,26) = 7.2, p<0.05) and a significant Group-by-Hemisphere interaction for Glx (F(1,24) = 4.6, p<0.05). In post-hoc ANCOVA covarying volume% gray and white matter, in right pACC only, Glx (9.5%, F(1,33) = 4.9, p<0.05), Cr (6.7%, F(1,35) = 6.5, p<0.05), and tNAA (10.2%, F(1,35) = 8.2, p<0.01) were significantly higher in the sample with ASD than in the control sample (Fig. 5). Glx was above the control mean for 16 of 23 subjects with ASD. Similar Glx elevation was observed when left and right pACC metabolite levels were averaged together. Upon removing subjects on medication, a notable Glx excess (8.5%) was still present in right pACC in the sample with ASD, though (perhaps due to the smaller number of subjects) this was no longer statistically significant. MRSI metabolite levels were also interrogated in left and right prefrontal white matter proximally lateral to the pACC in the same slab. In the sample with ASD, mean volume% white matter in these voxels was 86.3% ±4.6% (77.7–95.6%) on the left and 83.5% ±6.1% (74.3–96.2%) on the right; for the control sample, the values were 84.9% ±9.4% (70.0–99.5%) on the left and 79.9% ±5.6% (71.6–87.5%) on the right. There were no significant between-group differences in volume% white matter, gray matter, or CSF in these white-matter regions. In the sample with ASD, Glx in these voxels was 9.2±2.1 IU (6.0–13.5 IU) on the left and 9.7±1.4 IU (7.8–12.6 IU) on the right; for the control sample, the values were 9.5±0.9 IU (8.2–10.7 IU) on the left and 10.7±1.7 IU (8.5–13.2 IU) on the right. There were no significant between-group differences in Glx, Cr, tNAA or any other metabolite in these white-matter regions.

This multi-voxel MRS investigation of glutamatergic metabolites in pACC yielded three major findings in the right pACC only: 1) Glx was higher in subjects with ASD than in typically-developing control subjects, 2) Cr was higher in subjects with ASD, and 3) tNAA was higher in subjects with ASD. These findings reinforce the notion from Experiment 1 that pACC metabolism is abnormal in ASD, with hyperglutamatergia as one feature. Abnormalities in Cr and tNAA metabolism are less reliably supported. All three effects may be lateralized to the right pACC.

The first finding was elevated Glx in right pACC in ASD. As with the finding from Experiment 1 of elevated Glx in midline (left + right) pACC, this result supports hyperglutamatergic theories of ASD. All of the arguments advanced in the Discussion of Experiment 1 regarding the possible mechanisms and consequences of this hyperglutamatergia also apply here.

The second finding was elevated Cr in right pACC in ASD. As in the Discussion of the diminished Cr found in Experiment 1, this could be a sign of inefficient utilization of local cell energy, only with an aberration in the opposite direction in this instance.

The third finding was elevated tNAA in right pACC in ASD. An elevation in tNAA  =  NAA + NAAG may represent higher levels of NAA, of NAAG, or of both. Local elevations of NAA could result from multiple mechanisms, including faster astrocyte-membrane decomposition of NAAG into Glu and NAA [85], slower oligodendrocyte-membrane degradation of NAA into acetate and aspartate [103], [104], slower intraneuronal synthesis of NAAG out of NAA and Glu [105], and/or faster intraneuronal NAA synthesis out of aspartate and acetyl-CoA [103], [106]. Elevations of NAAG could result from faster pre-synaptic vesicular release [84], slower astrocytic decomposition into Glu and NAA, and faster neuronal synthesis out of NAA and Glu. Additionally, some genetic studies associate ASD with the SLC25A12 gene coding for the aspartate/glutamate carrier aralar1 in the mitochondrial membrane [19]–[23]. This carrier transports aspartate ions across the membrane out of the mitochondrion into the cytoplasm in exchange for Glu ions that leave the cytoplasm and enter the mitochondrion. Inside the mitochondrion, aspartate may be consumed by NAA synthesis and Glu may be consumed by the Krebs Cycle. Thus, any genetic variant that decreases transport efficiency and/or mitochondrial membrane density of aralar1 (if such be the case in ASD) could contribute to simultaneous elevation of Glx and tNAA, as seen in Experiment 2.

^1^H MRS has linked glucose metabolic rate to tNAA [107] and to Glx [108], while ^13^C MRS has linked glucose metabolic rate to NAA [109] and to Glu [110]. Among the factors underlying this linkage may be direct transport of water generated in energetic catabolism out of the neuron by NAA and NAAG [111], [112] and/or regulation of neuronal and glial water content by osmolytic NAA and Glu [87]. Thus, elevations of all three metabolites (Glx, Cr, and tNAA) may be associated with disturbed brain energy metabolism in ASD. The lack of significant between-group differences in the levels of these three metabolites in proximal prefrontal white matter suggests that the effects are characteristic of local cortex and not white matter.

Each of the three metabolite elevations was apparent only in the right pACC. These findings join prior neuroimaging reports of lateralized effects of ASD within the anterior cingulated [42], [53], [60]–[62], specifically to right anterior cingulate (as reviewed by Mundy) [72] in some cases. It is possible that, with more subjects, effects might be observed in both cerebral hemispheres.

Some subjects with ASD but no controls were receiving psychotropic medication at time of scan. MRSI voxels contained significantly more gray matter in subjects with ASD than in controls, although voxel tissue composition was covaried for and results of a targeted sampling of high white-matter voxels appeared to rule-out the possibility that observed effects were due to between-group differences in voxel tissue content.

## Discussion

The small pilot investigation, Experiment 1, indicated elevated Glx in pACC in ASD. A larger follow-up study, Experiment 2, in which the sample with ASD and the control sample were now better matched for gender and IQ again found elevated Glx in ASD, albeit restricted to right pACC. Thus, two separate studies using independent samples of children with ASD and controls and scanning on two different systems (GE, Siemens) both support the idea of pACC hyperglutamatergia in ASD.

As discussed in our review (Levitt et al., in press) [113], prior MRS studies reporting Glu or Glx in ASD have yielded mixed results, presumably due to differences in subject samples and MRS methods. Using rigorous MRSI methods, Friedman et al. (2003, 2006) [31], [32] found no effects of ASD on Glx in the cingulate. Unfortunately, these authors do not specify which subregion(s) of the cingulate they sampled. Also, all the subjects with ASD in their study underwent propofol sedation at time of scan, whereas that was the case for only one subject in Experiment 1 and for none in Experiment 2. Shulman et al. (2003, 2009) point out that brain energetic metabolism, and thence the rate of Glu-Gln cycling and thereby possibly Glx levels, is intimately related to anesthesia-induced level of consciousness [114], [115]. Finally, the subjects in Friedman et al. (2003, 2006) were considerably younger (<5 years old) than ours. Anatomic neuroimaging suggests differences between ASD in early and later childhood; for example, the well-known observation of brain overgrowth in ASD between 2–4 years [116], [117] that arrests or normalizes in later childhood and adolescence [118], [119]. Changes in regional neurometabolite levels in the developmental course of ASD may similarly underlie differences between our Glx findings and those of the Seattle Group. DeVito et al. (2007) [28] obtained below-normal Glx and tNAA diffusely in cortical gray matter in subjects with ASD. In particular, endpoint values were extrapolated from linear plots of metabolite levels sampled from voxels across entire cerebral lobes. Hence, it is not clear what levels of this metabolite were locally in pACC. Also, their TE of 135 ms leaves a rather small Glx signal that is perhaps less reliable than the signals of other metabolites. Sampling the amygdala-anterior hippocampus, Page et al. (2006) [47] measured elevated Glx in their sample with ASD. Experiments 1 and 2 are not strictly comparable with these prior studies as the latter did not specifically target the pACC using short-TE (25–30 ms) spectroscopy; to that extent, the present findings are novel. Using methods similar to our, Bernardi et al. (2011) [53] observed depressed Glx in right pACC, the opposite of the present findings, but in adult subjects with ASD. On the one hand, these opposite findings may reflect differences between adults and children. On the other hand, metabolite levels in our study are CSF-corrected and take account of MRSI voxel tissue composition while Bernardi et al. (2011) [53] do not provide this information. As metabolite levels vary between tissue types, it is possible that metabolite effects in Bernardi et al. (2011) [53] are confounded with intersubject variation in voxel tissue composition. Thus, while our studies of ASD offer anatomically highly targeted assays of pACC metabolite levels at short-TE accounting for voxel tissue composition, no previous study we are aware of offers comparable methodological rigor in the same age range (middle-childhood to adolescence).

Hence, we think that our two experiments add support to the notion that hyperglutamatergia exists at least in pACC and at least in middle to late childhood and adolescence in ASD. To determine whether this applies also in early childhood, a subsampling of the Seattle data within the pACC portion of their MRSI slab might be helpful; a more stringent and more challenging test would be to acquire from the region in non-anesthetized children, perhaps while sleeping or using very rapid pulse sequences. To determine whether hypo- or hyperglutamatergia applies in pACC in adults with ASD, results of an analysis of the Bernardi et al. (2011) data taking account of voxel tissue composition might be helpful.

The findings of hyperglutamatergia in our studies are generally consistent with recent broader theories of autistic spectrum disorders that emphasize an imbalance in cortical excitation vs. inhibition [120], [121]. Elevated glutamatergic metabolism, as seen in the present results, may be a consequence of deficient central GABAergic inhibition, as proposed in these theories. Based on human post-mortem and genetic and rodent ASD model investigations, inadequate GABAergic inhibition may have multiple developmental causes including underexpression of the GAD65 and GAD67 enzymes (that synthesize GABA from Glu), atypical GABA_A_ receptor subunit composition, and failure of neuroligin and neurexin synaptic binding proteins to appose post-synaptic GABA receptors precisely to presynaptic release sites. In the past few years, translational neuroscience has been making a mounting case for hypofunctioning of brain GABAergic systems ASD that could lead to excess brain glutamatergic metabolism.

Whereas Experiment 1 found diminished pACC Cr in the sample with ASD, Experiment 2 found elevated Cr in right pACC. Thus, abnormal Cr in pACC in ASD, if it does exist, is more variable and harder to reproduce than abnormal Glx. This variability might reflect unstable local cell energetics that induce an expanded creatine-phosphocreatine ATP buffer in some subjects and a contracted buffer in others. Page et al. (2006) [47] also observed elevated Cr in amygdala-hippocampus in ASD, and Levitt et al. (2003) [42] observed effects of ASD diagnosis on Cr in occipital cortex and caudate, so there is precedence for abnormal Cr in ASD, albeit in other brain regions.

Elevated tNAA was found in pACC in ASD in Experiment 2 only. Again, abnormal tNAA in ASD may be harder to reproduce than elevated Glx. In prior work, Oner et al. (2007) [46] registered higher tNAA/Cr and tNAA/Cho in right anterior cingulate cortex in subjects with Asperger's syndrome than in controls and Fujii et al. (2010) [33] found lower tNAA/Cr in anterior cingulate in subjects with autism than in controls. Interpretation of these results is partially obscured by normalization to Cr, which itself may vary, but they do suggest heterogeneous effects of ASD on tNAA. In other brain regions (reviewed in Levitt et al, in press) [113], investigators have often found below-normal tNAA or its ratios in ASD, although findings of above-normal and no difference also exist. How plausible is a local elevation of tNAA in the pACC? In addition to the above-cited MRS results, data from recent fMRI and hybrid fMRI-MRS experiments do, in fact, strongly suggest a special role for the pACC in ASD and autistic symptomatology. The pACC, for example, was one of the few brain regions demonstrating significant effects of ASD diagnosis in a recent metaanalysis of fMRI studies [66]. Working in healthy subjects, the same researchers related fMRI functional connectivity with the pACC with elevated levels of autistic traits [65]. Also in healthy controls, Duncan et al. (2011) [76] found correlations localized to pACC between MRS Glx and an fMRI effect related to subject empathy, low empathy being a common symptom of ASD. Finally, elevated intensity was observed in at-risk carriers of an autism-associated CNTNAP2 allele in pACC [75]. These and other neuroimaging results give ample evidence for focal effects of ASD diagnosis and autistic traits and autistic symptoms in the pACC. It is therefore not surprising to find MRS metabolic effects particular to that brain region.

Experiment 2 alleviated several but not all limitations of Experiment 1. Both studies were still conducted at low-field (1.5 T) and expressed their results as Glx rather than as Glu and Gln separately. Based on low field strength and, in the case of MRSI, small voxel size, our quality control procedures used the standard 20% SD criterion of the LCModel fitting package and a SNR cut-off of 3 for MRSI and 5 for single-voxel MRS. Although some spectroscopists might prefer stricter cut-offs, working with these values we found that individual metabolite peaks were typically readily identified by eye and easily fit by automated routines. Also single-subject data quality was frequently higher than the cut-off values. In neither study was it possible to match between-group voxel tissue-composition thoroughly (although differences were dealt with through statistical covariates and co-sampling proximal white matter). Efforts to match tissue composition may have been aggravated by putative effects of ASD on anterior cingulate cortical volume or thickness [58]–[61]. Future MRS and MRSI studies at 3 T will allow smaller, hopefully more tissue-pure voxels and also better spectral segregation of Glu and Gln. Regarding the latter, better segregation might also be achieved by acquiring spectra at TE  = 80 ms, thought to be optimal for quantifying Glu [122]–[124]. Future investigations should also include MR relaxation studies, as autism may affect metabolite and water relaxation times [31], [125]. Finally, in both Experiments, several subjects with ASD were undergoing treatment with psychotropic medication at time of scan. Ideally, one would test only drug-naïve subjects, although, given clinical realities, this can be difficult to achieve on a practical time scale. These limitations notwithstanding, the present findings suggest that Glx is elevated locally in pregenual anterior cingulate cortex in subjects with ASD. This elevation, combined with less certain effects of autism on Cr and tNAA may reflect disturbances of cell-energy metabolism.
